# Prevalence of Raynaud Phenomenon and Nailfold Capillaroscopic Abnormalities in Fabry Disease: A Cross-Sectional Study

**DOI:** 10.1097/MD.0000000000000780

**Published:** 2015-05-22

**Authors:** Samuel Deshayes, Laurent Auboire, Roland Jaussaud, Olivier Lidove, Jean-Jacques Parienti, Nathalie Triclin, Bernard Imbert, Boris Bienvenu, Achille Aouba

**Affiliations:** From the Departments of Internal Medicine (SD, BB) and Biostatistics (JJP), C.H.U. Côte de Nacre, Caen; University of Tours, Inserm Imagerie et Cerveau UMR U930, Tours (LA); Department of Internal Medicine and Infectious Diseases, C.H.U. Robert Debré, Reims, France (RJ); Department of Internal Medicine, Hôpital de la Croix Saint-Simon, Paris (OL); APMF, Vendresse (NT); Department of Internal Medicine, C.H.U. Michallon, Grenoble, France (BI).

## Abstract

Fabry disease (FD) is a lysosomal disorder leading to progressive systemic involvement, including microvascular damage that leads to neurological and cardiovascular disorders. We hypothesize that the latter could be documented at an early stage by performing a microcirculation study with nailfold capillaroscopy and evaluation of Raynaud phenomenon.

The objective was to measure the prevalence of Raynaud phenomenon and nailfold capillaroscopic abnormalities in FD.

This cross-sectional study included a standardized questionnaire and a nailfold capillaroscopy that assessed previously reported patterns in FD (dystrophic and giant capillaries, avascular fields, irregular architecture, dilatation and density of capillaries, hemorrhage), and was conducted on 32 Fabry patients and 39 controls. Capillaroscopic photographs were reviewed by 2 independent blinded investigators.

Twelve Fabry patients (38%) suffered from Raynaud phenomenon, 5 were males (ie, 50% of male Fabry patients), compared with 2 controls (13%) (*P* < 0.001), of whom none were males (*P* < 0.001). Raynaud phenomenon was concomitant or before the occurrence of pain in the extremities in 42% of Fabry patients.

More ramified capillaries were significantly observed in Fabry patients (12/32, 38%) than in controls (5/39, 13%, *P* = 0.016).

Secondary Raynaud phenomenon should lead to screening for FD, especially in men. By extension, in high-risk populations for FD, the presence of Raynaud phenomenon and ramified capillaries should be assessed.

## INTRODUCTION

Fabry disease (FD), secondary to a deficit in α-galactosidase A caused by abnormalities in the *GLA* gene, belongs to X-linked lysosomal disorders. The progressive accumulation of neutral glycosphingolipids, particularly globotriaosylceramide, within lysosomes leads to a multiorgan disease.^1,2^ The “classic” severe phenotype, including systemic involvement, has an estimated incidence of 1 in 117,000 in the general population.^3^ This figure is probably underestimated, as suggested by newborn screening and by the discovery of atypical variants with late-onset isolated renal or cardiac manifestations.^4–7^ A diagnosis is frequently delayed for many years because of non-specific signs.^8^

Enzyme-replacement therapy is available and decreases morbidity,^9–11^ especially if it is started before the onset of complications, hence the importance of early screening of these patients. One of the earliest and common symptoms is pain in the extremities,^1^ classically attributed to neurologic involvement (small nerve fibers of the peripheral somatic and autonomic nerve systems).^12–14^ FD also induces vasculopathy of small vessels by accumulation of neutral glycosphingolipids in smooth muscular and endothelial cells, which affect the microcirculation.^15,16^ Raynaud phenomenon results from a disorder of vascular thermoregulatory control mechanisms, related to an increased sympathetic activity affecting the microcirculation and to endothelial dysfunction. This is more pronounced in the case of secondary Raynaud phenomenon.^17^

Although nailfold capillaroscopy assesses small vessels and Raynaud phenomenon is secondary to dysregulation of vascular homeostasis, little research has been done on these subjects in FD.

The first identification of dystrophic capillaries in FD occurred in 1993 with the description of 3 case reports,^18^ followed by another case report of ramified capillaries in 1994,^19^ and a study of 8 members of a family who had FD in 2006, which reported 37% of ramified capillaries.^20^ Wasik et al,^21^ in a matched case–control study with 25 Fabry patients (17 men, 8 women) in 2009, reported 72% of dystrophic capillaries, with a higher incidence of Raynaud phenomenon in Fabry patients, especially male patients (20%). Costanzo et al,^22^ in 2014, supported these results and reported 52.6% of dystrophic capillaries in a study of 19 Fabry patients, compared with 0% in 19 matched controls.

The objective of this study was to measure the prevalence of Raynaud phenomenon and nailfold capillaroscopic abnormalities in a larger cohort of Fabry patients.

## METHODS

### Patients

We performed a cross-sectional study during a patient-support association meeting. This meeting, organized by the FD French patients association in June 2013, brought together already diagnosed Fabry patients from all over France. Diagnoses were made by experienced clinicians in FD on patients presenting clinical signs or familial history of the disease with a biological confirmation (α-galactosidase activity with a genetic analysis of the α-galactosidase A gene for men, and excretion of urinary Gb3, α-galactosidase activity, and gene mutation analysis for women). The subjects fulfilled the following inclusion criteria: being aged ≥18 years, and had given their written informed consent after oral information was supplied by the investigators.

### Data Collection

The subjects viewed a slideshow focusing on Raynaud phenomenon, describing its 3 phases with photographs. They then completed a standardized self-assessment questionnaire, including age, gender, medical history, cardiovascular risk factors, and manifestations of FD, enzyme-replacement therapy, and Raynaud phenomenon. They also underwent a nailfold capillaroscopy^23^ of the 3 last fingers of each hand (CapXview HD, Xport technologies, Craponne, France) to assess the following parameters, according to the previously reported patterns observed in FD:

1. dystrophic capillaries (particularly bushy and ramified capillaries),
2. avascular fields,
3. irregular architecture,
4. dilatation of capillaries,
5. giant capillaries,
6. hemorrhage, and
7. density of capillaries (per mm).

Two independent blinded reviewers assessed the capillaroscopic photographs. A consensus was reached regarding any differences. Accompanying persons constituted the control group.

This study was approved by the local ethics committee.

Data sets are available from the Dryad repository, at http://datadryad.org/ with the doi:10.5061/dryad.kq04t.

### Data Analyses

Statistical analyses were performed using R 3.0.3 software. Categorical variables are reported as percentages and were compared using the χ^2^ or Fisher exact test, according to expected frequencies. Continuous variables are expressed as their means ± standard deviations, and were analyzed using Student *t* test. A *P* value of <0.05 was considered statistically significant.

## RESULTS

**FIGURE 1:**
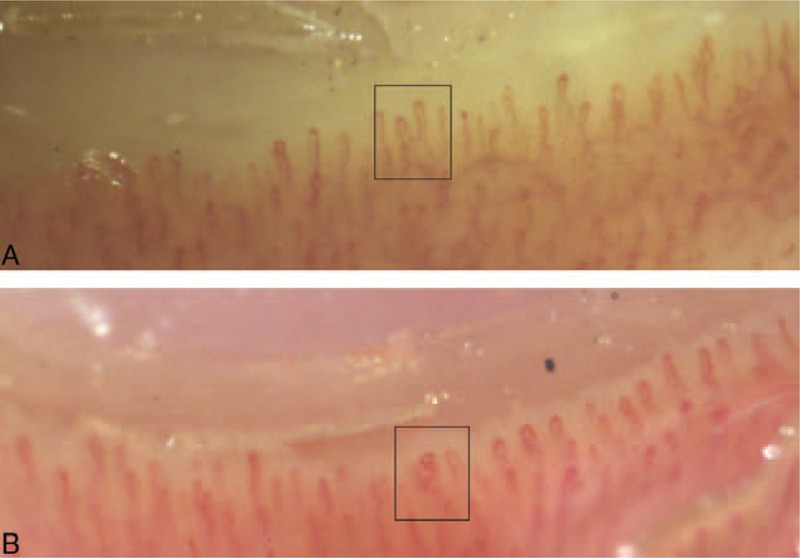
Capillaroscopic photographs of (A) ramified and (B) bushy capillaries (×50).

Seventy-one questionnaires were collected from 32 Fabry patients (45%) and 39 controls (55%). Concerning demographic data, sex ratio was statistically different, with more men in the control group (Table 1). Data regarding Raynaud phenomenon are presented in Table 2. Pain in the extremities was reported among 28 Fabry patients (88%) versus in none of the controls (*p* < 0.001). Patients with FD and Raynaud phenomenon all suffered from pain in the extremities, versus no pain in the controls (*P* = 0.011). Raynaud phenomenon was concomitant or before the occurrence of pain in the extremities in 42% of Fabry patients. Twenty-five of the 32 Fabry patients (78%) (10/10 males, 15/22 females) were receiving enzyme-replacement therapy. The only nailfold capillaroscopy parameter that was statistically different was a particular major dystrophic capillary pattern (Table 3, Figure 1).

**TABLE 1:**
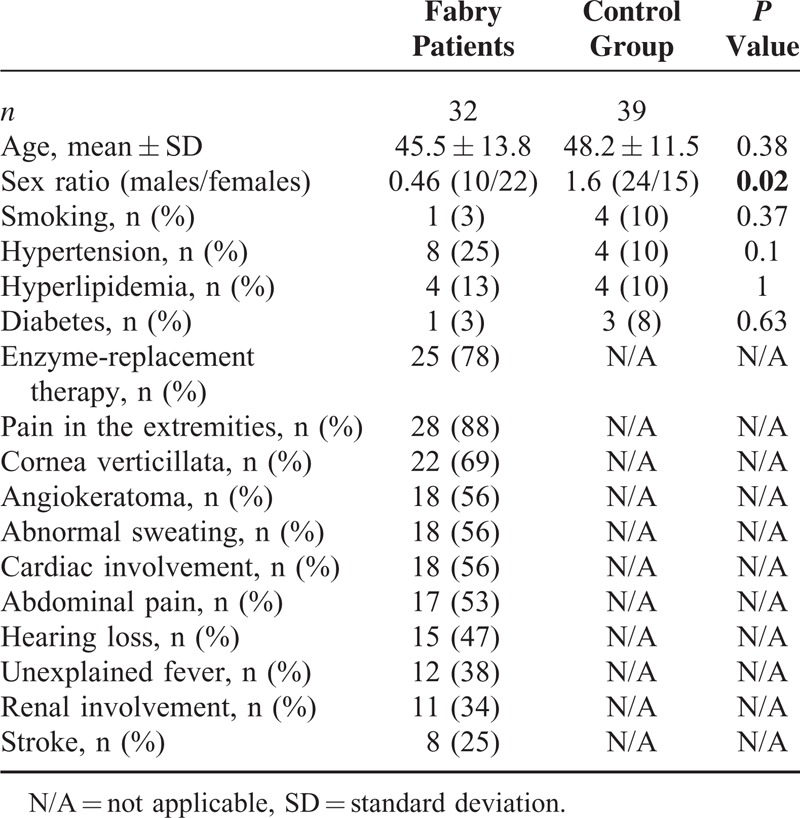
Demographic and Clinical Characteristics of Fabry Patients and Controls

**TABLE 2:**
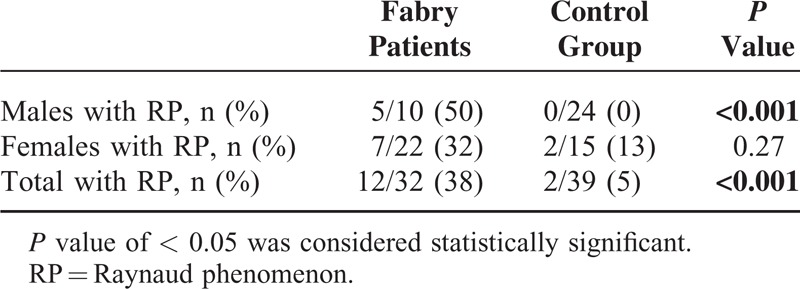
Characteristics of Patients Suffering From RP

**TABLE 3:**
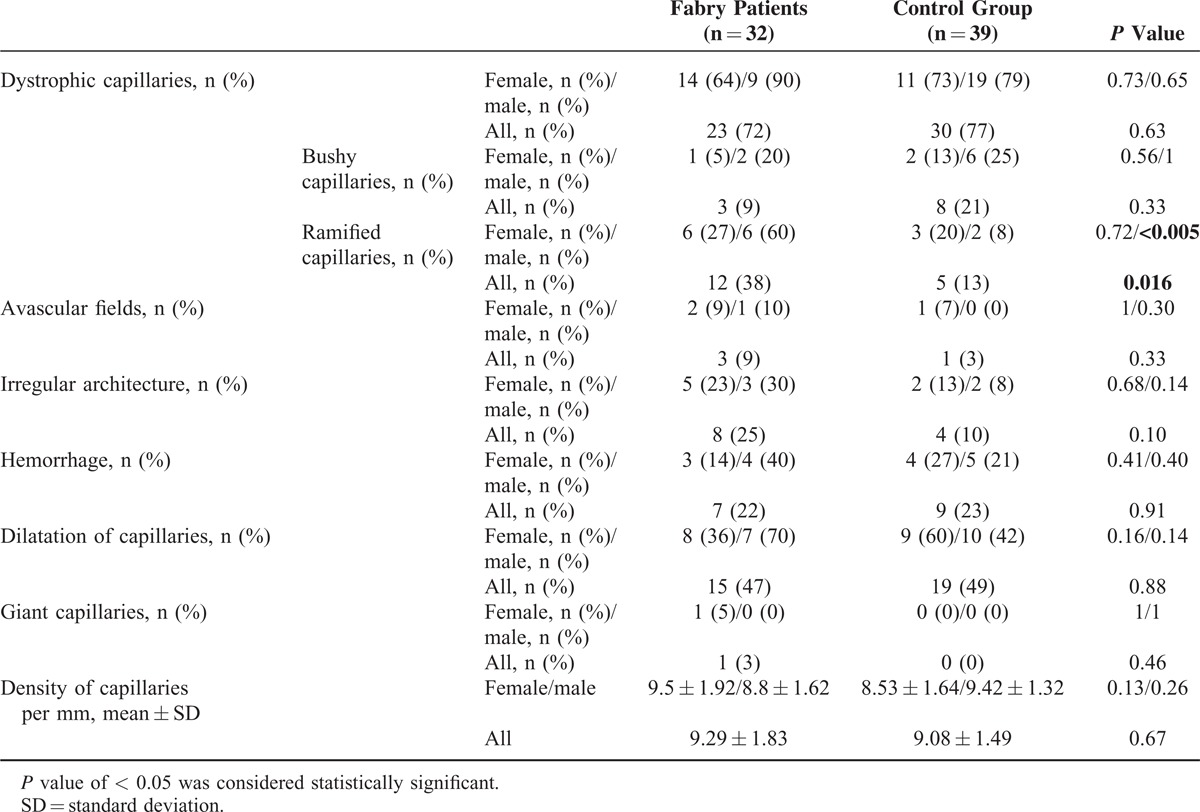
Results of Nailfold Capillaroscopic Parameters

## DISCUSSION

This study reveals a higher incidence of Raynaud phenomenon in Fabry patients (38%) than in controls (5%), versus 3–22%^24–26^ in the general population. This figure is higher than the 15.3% reported in the study by Germain et al, who measured the prevalence of Raynaud phenomenon in Fabry patients.^27^ Among males, half of our Fabry patients had Raynaud phenomenon compared with none among the control group. According to the literature, the prevalence of Raynaud phenomenon varies from 0.5 to 16%^26,28,29^ in the general male population, and 2.5% to 22% in the general female population.^26^

Secondary Raynaud phenomenon in men is usually thought to be associated with chronic occupational exposure, especially in manual workers, which may explain the higher incidence of dystrophic capillaries in men.^30^ The high prevalence of Raynaud– phenomenon in FD, hardly described so far, should be considered as a cause of secondary Raynaud phenomenon. By inducing small-fiber neuropathy (both autonomic and sensitive),^12,13^ endothelial dysfunction,^15,16,31^ smooth-muscle-cell proliferation,^32^ the accumulation of neutral glycosphingolipids may impair vascular tone and favor Raynaud phenomenon. Because Raynaud phenomenon is concomitant or before the occurrence of pain in the extremities in almost half of Fabry patients, this could be, at least in part, a causal factor of this pain.

Consistent with data in the literature,^18–22^ a significantly higher rate of ramified capillaries in Fabry patients was observed, particularly in males, which may reflect a more severe involvement of the microcirculation in the latter, as already identified for other involvements.^1^ In contrast, no significant difference was found regarding dystrophic capillaries as a whole. This discrepancy might be explained by the higher proportion of males, more susceptible to dystrophic capillaries,^30^ within our control group compared with the Fabry group, or it may have been because of improved microcirculation after treatment. Indeed, 78% of our Fabry patients were receiving enzyme-replacement therapy, compared with 0% in the other 2 published studies. López-Rodríguez et al^20^, in their study of a family of 8 members who had FD (5 of them receiving enzyme-replacement therapy), noted that the 3 patients with a normal nailfold capillaroscopy were under treatment. It would be interesting to carry out a prospective study on the diagnostics of FD to monitor any associations between capillaroscopic abnormalities and enzyme-replacement therapy.

Angiokeratoma and tortuosity of the conjunctival and retinal blood vessels have been previously described.^1^ It now seems that dystrophic capillaries, observed by nailfold capillaroscopy, and newly reported vessel tortuosity, visible on the external surface of the upper eyelid,^33^ enrich Fabry vasculopathy of new clinical signs that can be easily accessed in a clinical examination. These clinical signs could improve screening of people in high-risk populations for FD (hypertrophic cardiomyopathy, dialysis patients, stroke in young people).^1^

To our knowledge, this study is the third,^21,22^ but the largest, study to assess nailfold capillaroscopic abnormalities in FD. The control group was constituted of family members, sharing the same genetic and/or environmental factors, potential source of confounding factors. It was not matched on age and sex. Despite this, age was not statistically different between the 2 groups. The female predominance in our Fabry patients can be explained by a higher mortality and morbidity rate in male patients before the availability of enzyme-replacement therapy,^34^ impeding their participation to meetings, and/or by a possible over-representation of females in the patient-support association meetings.^35,36^ This non-comparability of the control group regarding sex ratio is the main limitation of this study. Nevertheless, the predominance of males in the control group, more susceptible to dystrophic capillaries, could have revealed a more specific pattern of dystrophic capillaries in FD, namely ramified capillaries, not discovered hitherto. Moreover, the inhomogeneity of Fabry patients regarding enzyme-replacement therapy, unlike the other 2 studies, opens up new perspectives of studies that can explain some of the discrepancies encountered with previous data. Because this study was based on self-assessment questionnaire, no data on mutations were available. Therefore, no genophenotypic correlation could be done. We report the prevalence of Raynaud phenomenon and nailfold capillaroscopic abnormalities in French Fabry patients. Hence, our findings may not apply to other countries with different genetic and environmental factors.

## CONCLUSION

Our data strengthen the fact that secondary Raynaud phenomenon should lead to screening for FD, especially in men. By extension, in high-risk populations for FD (ie, hypertrophic cardiomyopathy, dialysis patients, stroke in young people), the presence of ramified capillaries and Raynaud phenomenon should also be assessed.
